# Pre-treatment T-cell Transcriptional Signatures Predict Immunotherapy Outcomes in Melanoma

**DOI:** 10.21203/rs.3.rs-10629841/v1

**Published:** 2026-08-25

**Authors:** Noah Lepola, Caroline Dravillas, Shannon Gray, Michael S. Bodnar, Namrata Arya, Richard Wu, Claire Verschraegen, William E Carson, Kari L Kendra, Daniel J Spakowicz, Christin E Burd

**Affiliations:** The Ohio State University; The Ohio State University; The Ohio State University; The Ohio State University; The Ohio State University; The Ohio State University; The Ohio State University; The Ohio State University; The Ohio State University; The Ohio State University; The Ohio State University

## Abstract

Immune checkpoint inhibitors (ICIs) have improved outcomes for patients with melanoma and are now the standard of care for high-risk and advanced disease. However, long-term benefits are observed in only around 25% of patients, with significant risk for immune-related adverse events, highlighting the need for predictive biomarkers. To develop a minimally invasive, pre-treatment biomarker strategy, we profiled functional and subset-specific transcripts in peripheral blood T lymphocytes (PBTLs) and applied machine learning to identify predictive signatures. Patients were enrolled prior to receiving ICI monotherapy in the adjuvant (Exploratory n=61, Validation=78) or metastatic (Exploratory n=48, Validation=46) settings. Following feature selection, random forest models were trained and benchmarked against empirical null models. In the adjuvant setting, CD160 and GZMB predicted recurrence (95th percentile), while treatment-limiting toxicity was predicted by a signature comprising TNFRSF18, VTCN1, TIGIT, CCR4, and AHR (97th percentile). In the metastatic setting, baseline CD45RB, a marker of T-cell differentiation, most strongly predicted progression within one year (93.9th percentile). Distinct signatures in the adjuvant and metastatic settings suggest differences in T-cell programs associated with patient outcomes. These findings support further evaluation of pre-treatment circulating T-cell transcriptional profiles as predictors of ICI response and toxicity in melanoma.

## Introduction

Immune checkpoint inhibitors (ICIs) have transformed the therapeutic landscape of melanoma, yielding substantial survival benefits for patients with locally advanced and metastatic disease. Across multiple clinical trials, ICI monotherapy has extended median overall survival in metastatic melanoma by up to three years [1–5]. Comparable gains are observed in earlier-stage disease, where adjuvant ICI monotherapy confers 3.5- to 4-year recurrence-free survival rates in 50–65% of patients, with overall survival approaching 80% in some cohorts [6–12].

Despite these advances, treatment remains limited by both toxicity and incomplete efficacy. Serious immune-related adverse events (irAEs), classified as grade III or above, occur in approximately 15-20% of patients receiving ICI monotherapy and can lead to treatment discontinuation and, in some cases, persistent morbidity or death [3, 13, 14]. These risks are particularly consequential in the adjuvant setting, where patients are clinically disease-free following surgical resection. In a randomized study of resectable stage III melanoma, nearly half of patients in the placebo arm remained recurrence-free at 3.5 years, suggesting that a substantial proportion may not require systemic therapy for long-term remission [11]. However, recurrence still occurs in a significant subset of treated patients, with 34.7% experiencing relapses despite adjuvant ICI therapy [11]. Therefore, many patients are either overtreated or fail to benefit from ICI therapy. More accurate predictive biomarkers will help guide patient treatment, particularly in the adjuvant setting.

The identification of predictive biomarkers of ICI response and toxicity remains an active area of research, but few candidates meet the threshold of predictive performance or supporting evidence required for clinical use. Among the most extensively studied markers, lactate dehydrogenase (LDH) has been incorporated into AJCC staging for melanoma, with elevated levels reflecting advanced disease and inferior clinical outcomes [15]. Meta-analyses in advanced melanoma have shown that elevated LDH is associated with worse overall survival among patients receiving ICI therapy (hazard ratios of 1.72-2.44); however, the predictive value of LDH in the adjuvant setting remains poorly defined [16, 17]. Consequently, LDH is not currently recommended for use in guiding ICI treatment [18, 19]. Another potential marker is the expression level of programmed death-ligand 1 (PD-L1) on tumor cells, based on the premise that higher levels may identify patients more likely to benefit from ICI therapy. However, clinical trial data have shown inconsistent associations with ICI response, limiting the utility of PD-L1 for treatment stratification in melanoma [2, 18–20]. Additional biomarkers, including tumor-infiltrating lymphocyte counts, gut microbiome composition, tumor-derived microRNA signatures, and circulating tumor DNA, show early promise but lack sufficient validation for clinical implementation [18–27]. Accordingly, no biomarkers are currently recommended by NCCN or ESMO guidelines to guide the use of non-targeted ICI therapy.

The identification of clinically relevant biomarkers is increasingly driven by machine learning (ML) approaches, which enable the integration and analysis of high-dimensional datasets at a scale that is not practical using conventional methods. ML algorithms can systematically uncover complex, multivariate relationships in an evidence-based and minimally biased manner, making them particularly well-suited for biomarker discovery. Among these, random forest (RF) has emerged as a widely utilized algorithm due to its ability to handle large data sets, resilience against outliers, robustness to input data distributions, and interpretability.

Blood-based biomarkers offer several practical advantages over tissue-based approaches, including minimally invasive sample collection with reduced procedural risk and broader feasibility across clinical settings. T cells are also critical for ICI therapies, so the ability to easily assess the general state and functionality with a blood test would be a useful tool. In addition, such assays do not require tumor tissue and can be implemented using standardized workflows that minimize the need for specialized interpretation, as is often required for tumor tissue-based measures, such as immunohistochemistry.

In this study, we evaluated whether transcriptomic profiles of circulating peripheral blood T lymphocytes (PBTLs) could predict patient outcomes in both the adjuvant and metastatic melanoma settings. We used the NanoString platform for targeted transcriptomic profiling, which enables rapid, reproducible measurement of predefined gene expression signatures without the technological and bioinformatic requirements of RNA-seq. The custom OSU_Senescence CodeSet was designed to characterize T cell phenotypes and functional states. The panel includes transcripts that distinguish major T cell subsets (e.g., cytotoxic and regulatory T cells) and serve as surrogate measures of immune activation and inhibition, cytokine production, effector function, and cell cycle regulation [28]. PBTLs were selected for analysis based on the central role of T cells in mediating response to ICI therapies.

Pre-treatment transcript levels measured by OSU_Senescence were incorporated into RF models to predict patient outcomes, and model performance was evaluated in validation cohorts of patients set to receive adjuvant or metastatic ICI monotherapy. Using this approach, we generated parsimonious models comprised of a minimum number of transcripts that predicted patient outcomes with significantly better performance than models derived from clinical parameters alone.

## Methods

### Patient Cohorts

We recruited adult patients (≥18 years) from the Cutaneous Oncology Clinic at The Ohio State University James Comprehensive Cancer Center under biobanking protocols 1999C0348 and 2017C0054. Patients had metastatic melanoma or had undergone complete melanoma resection and were scheduled to receive pembrolizumab or nivolumab, with all therapies administered according to standard-of-care guidelines. Patients scheduled to receive adjuvant ipilimumab monotherapy were also recruited but were excluded from this analysis to avoid confounding.

Participants in the adjuvant study were enrolled in two phases: an exploratory cohort (n = 50; 2016–2019) and a validation cohort (n = 59; 2019–2021). The metastatic study followed the same design, with an exploratory cohort (n = 48; 2016–2018) and a validation cohort (n = 46; 2018–2021).

Participant age, smoking history, sex, primary tumor type, and staging were abstracted from electronic medical records, physician notes, and pathology reports of biopsied tumor specimens. LDH levels were measured on the day of therapy initiation. Patient medical records were followed until the prescribed therapy was completed, or for one year.

Reasons for premature therapeutic discontinuation, including recurrence or progression, toxicity, patient preference, or physician discretion, were documented. In the adjuvant setting, recurrence was defined as evidence of disease during routine follow-up. In the metastatic setting, progression was defined as a 20% or greater increase in diameters across all lesions and a minimum increase of 5 mm in the sum, as determined by RECIST 1.1 criteria [29]. Discontinuation due to toxicity was defined as an adverse event resulting in a change of treatment or therapeutic hold exceeding three months in duration.

### Sample collection and peripheral blood T cell isolation (PBTL)

Peripheral blood samples were collected shortly before treatment, with all samples used in the machine learning models obtained on the day of treatment initiation. CD3+ peripheral blood T cells (PBTLs) were isolated from whole blood via negative selection with the EasySep Direct Human T Cell Isolation Kit (StemCell Technologies #19661). Purified PBTLs were resuspended in Qiagen RNA lysis buffer, flash frozen, stored at −80°C until RNA extraction using the RNeasy Plus Mini kit (Qiagen #74134).

### Nanostring mRNA quantification

A custom Nanostring CodeSet, OSU_Senescence, was used to measure five internal controls (*GUSB, HPRT1, PGK1, UBC, YWHAZ*), fourteen mRNAs associated with T cell subsets (*AHR, BCL6, CD127, EOMES, FOXO1, FOXP3, GATA3, PRDM1, RORA, RORC, RUNX3, SPI1, TBX21, ZBTB7B*), eight associated with T cell surface markers (*CCR4, CCR6, CD45RO, CD45RA, CD45RB, CXCR3, IL2RA, TNFRSF18*), six associated with T cell receptors and coreceptors (*CD3D, CD3E, CD4, CD8A, CD27, CD28*), seven associated with cell cycle regulation (*B3GAT1, CDKN1B, CDKN2A (ARF), CDKN2A (p16), EGR1, IL6, KLRG1*), eleven cytokines (*GZMB, IFNG, IL2, IL4, IL10, IL17A, IL21, ITCH, RNF128, TGFB1, TNF*), and twenty eight co-stimulatory and inhibitory receptors (*ADORA2A, BTLA, CD80, CD86, CD96, CD160, CD200R1, CD244, CD274, CD276, CEACAM1, CTLA4, HAVCR2, KIR Group 1 (act.), KIR Group 2 (act.), KIR Group 1 (inh.), KIR Group 2 (inh.), LAG3, NECTIN2, PDCD1, PDCD1LG2, PVR, PVRIG, SPN, TIGIT, TNFRSF14, VSIR, VTCN1*).

PBTL RNA integrity was verified on an Agilent TapeStation, and cDNA synthesized with SuperScript VILO Master Mix (Life Technologies). To detect low-abundance transcripts, including *CD244*, *CD274*, *CD276*, *CDKN2A* (*p14^ARF^*), *CDKN2A* (*p16^INK4a^*), *IL6*, and *IL17A*, samples were pre-amplified as previously described using custom Nanostring primers and TaqMan PreAmp Master Mix^26^. Raw data were normalized to five internal controls and analyzed using nSolver software prior to log_2_ transformation.

### Data analysis

The tableone package (v0.13.2) was used to compare clinical and demographic characteristics between cohorts using Student’s t-tests for continuous variables and chi-squared tests for categorical variables [30]. Differences were considered significant at *p* < 0.05.

Logistic regression–based feature selection was conducted using a generalized linear model with a binomial distribution and a logit link in the R stats package (v4.4.0) to identify OSU_Senescence mRNAs associated with treatment completion, toxicity-related discontinuation, or disease recurrence/progression [31]. P-values were calculated using the Wald test and used to determine the order in which mRNAs were incorporated into the limited-dataset machine learning models. Alternatively, features were selected using Boruta (v9.0.0) [32]. Twenty-five runs of Boruta were performed, and transcripts that appeared in 13 or more runs were utilized in a predictive model. RF models were built using the randomForest package in R (v4.7-1.1), with performance assessed against 1,000 outcome-shuffled models that preserved class proportions [33]. F1 score, the harmonic mean of precision and recall, was selected as the output metric due to dataset imbalance.

All models were trained on exploratory cohorts and tested on validation cohorts. For comparisons between the adjuvant and metastatic settings, models were trained on the exploratory cohort from one setting and tested on the validation cohort from the other setting.

## Results

### Adjuvant cohort characteristics

**Figure 1:**
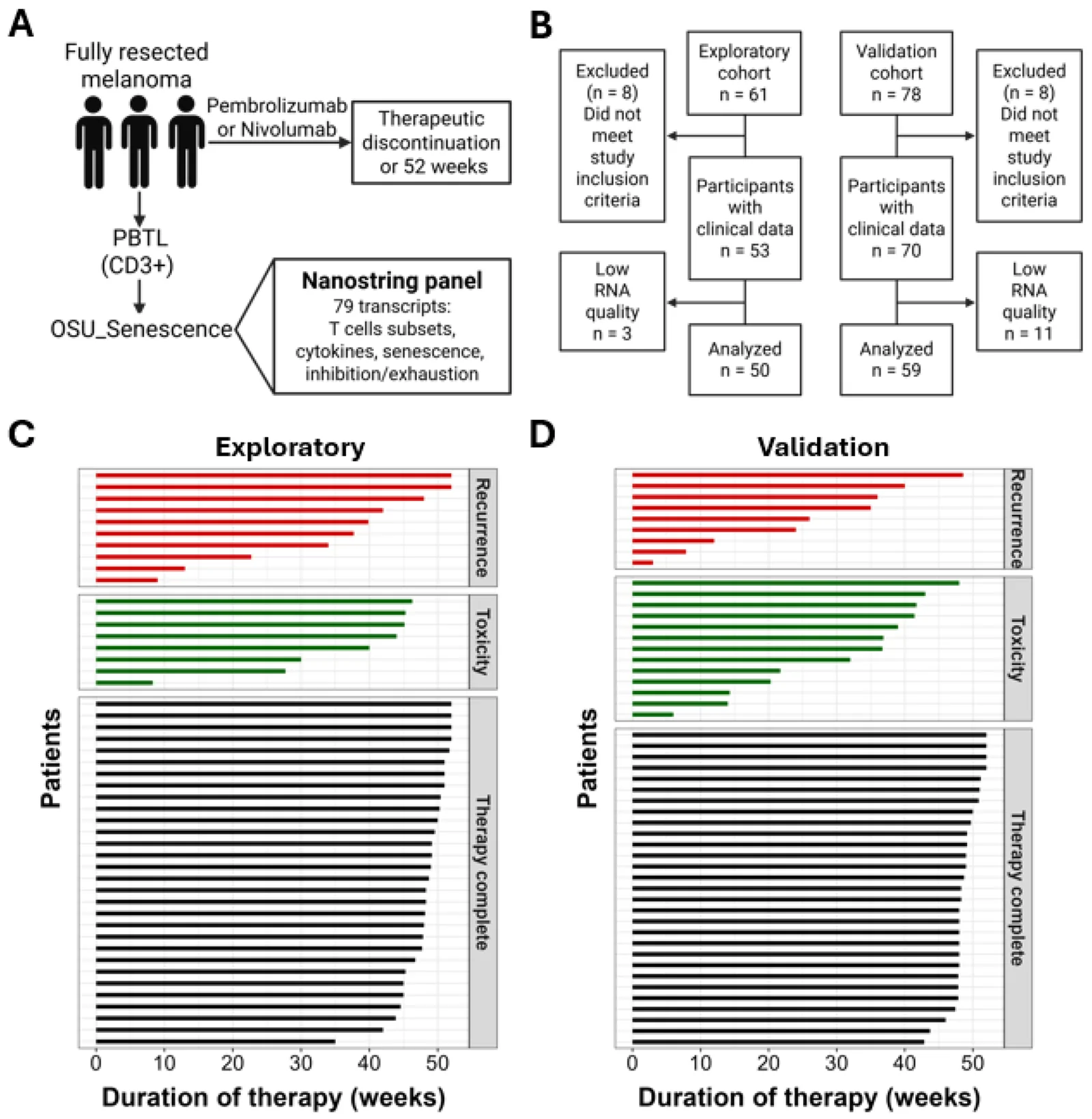
Study overview and patient timelines for adjuvant cohorts. (A) Overview of study timeline and measured outcomes. Patients with fully resected melanoma were enrolled prior to the initiation of adjuvant immune checkpoint inhibitor (ICI) monotherapy. Baseline peripheral blood T lymphocytes (PBTL) were profiled utilizing the OSU_Senescence panel. Transcriptal, clinical, and outcome data were then used to train predictive models. (B) CONSORT-style flow diagram depicting patient recruitment to the exploratory and validation cohorts and reasons for exclusion from the final analysis. (C, D) Treatment timelines for patients in the exploratory (C) and validation (D) cohorts, grouped according to clinical outcome.

Patients with fully resected melanoma who were prescribed adjuvant immune checkpoint inhibitor (ICI) therapy were approached prior to treatment initiation (Figure 1A). A total of 61 patients were enrolled in the exploratory cohort and 78 in the validation cohort (Figure 1B). Eight participants in each cohort were excluded from further analysis due to receipt of combination therapy or a revised diagnosis. Peripheral blood was collected prior to therapeutic initiation, and CD3^+^ peripheral blood T lymphocytes (PBTLs) were isolated for RNA extraction and subsequent analysis on the OSU_Senescence panel. Patients were prospectively followed for the duration of therapy or up to one year (Figure 1C–D).

The adjuvant exploratory and validation cohorts were comparable across baseline characteristics, with treatment being the primary differentiating feature (Table 1). This distinction was driven by a greater use of pembrolizumab in the exploratory cohort, reflecting evolving prescribing practices over the recruitment period. In both cohorts, males outnumbered females, consistent with historic melanoma trends in the US[34]. Most patients were diagnosed with stage III disease (Exploratory 74%, Validation 79.7%), consistent with the population most commonly eligible for adjuvant immunotherapy. Discontinuation due to therapy completion was the most common outcome in both cohorts (Exploratory 60%, Validation 49.2%). Most patients completed therapy (Y%).

### *CD160* and *GZMB* transcript levels predict recurrence in adjuvant melanoma

**Figure 2:**
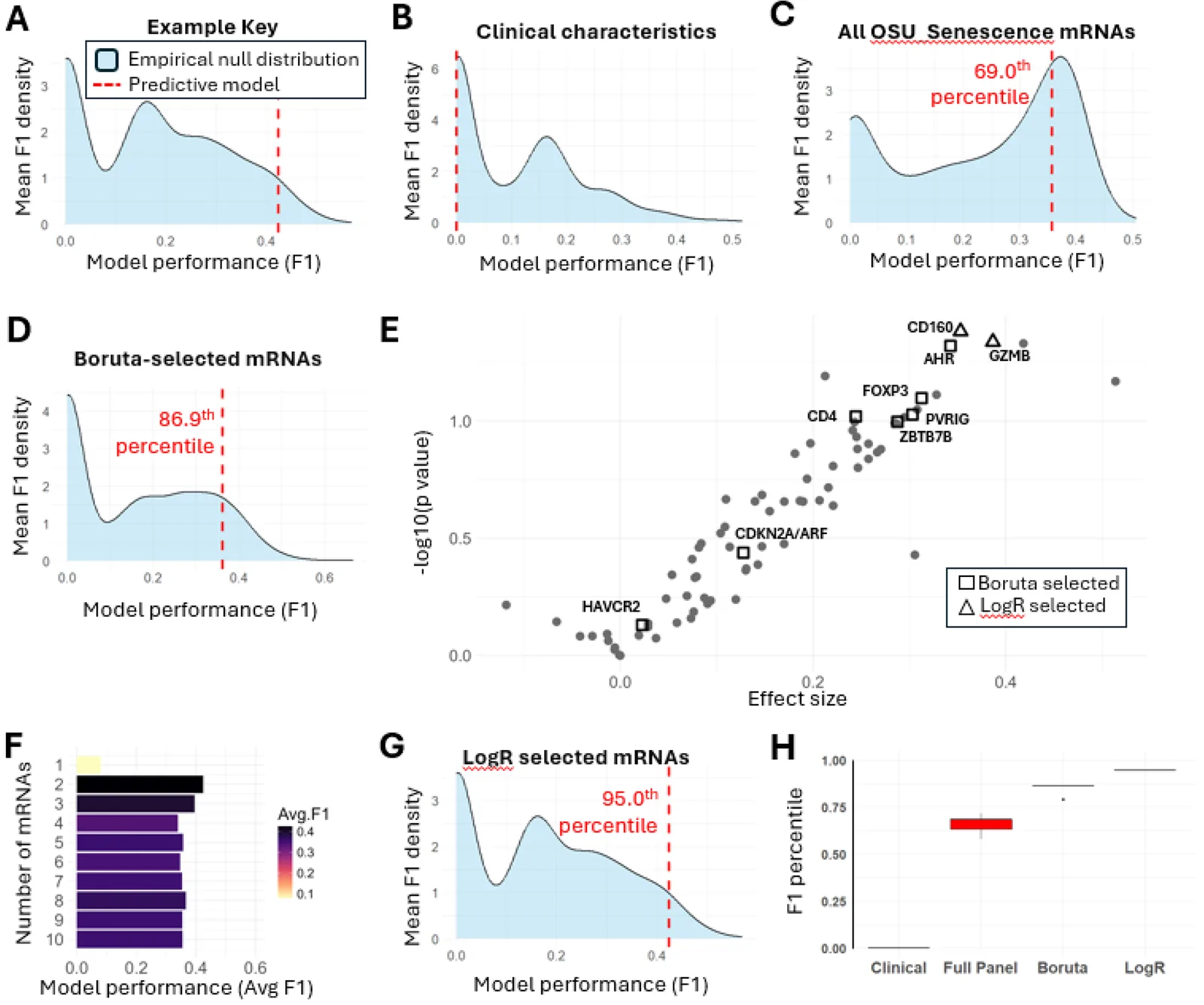
Baseline PBTL CD160 and GZMB levels outperform empirical null model when predicting recurrence in the adjuvant setting. (A) Representative diagram of model performance. Empirical null models are represented by blue curves which show F1 score distributions from 1,000 random forest models utilizing datasets with randomly permuted patient outcomes. Predictive models are represented by a dashed red line, which indicates the mean performance of models trained on the original, unpermuted dataset across 25 runs. (B-D) Models as described in (A) based on clinical characteristics (B), all 79 mRNAs measured by the OSU_Senescence panel (C), or the seven mRNAs identified as key features by Boruta (D). (E) Logistic regression of OSU_Senescence transcripts by patient outcome, with position on x axis indicating higher expression in patients completing therapy (right) versus those experiencing recurrence (left). (F) Mean F1 scores (25 runs) for random forest models predicting melanoma recurrence, with mRNAs sequentially incorporated according to increasing p-value (panel D). (G) Model as described in (A) using the two mRNAs identified in (E) (CD160 and GZMB). (H) Percentile distribution of predictive model F1 scores from (A), (B), (C), and (D) respectively, and were calculated by comparing F1 score of predictive models to the distribution of F1 scores from the empirical null models.

We evaluated four approaches to develop and optimize models for predicting melanoma recurrence in the adjuvant setting: clinical characteristics, full transcript panel, transcript selection by Boruta, and transcript selection by logistic regression. Models were trained using the exploratory cohort and tested on the validation cohort. To evaluate the models, we selected the F1 score over other performance metrics to account for the imbalance in patient outcomes (completed vs. prematurely discontinued treatment) and to provide a unified metric to integrate precision and recall, both of which are clinically relevant in this context. To contextualize the models’ performance, we created an empirical null model for every outcome by randomly permuting the outcome labels for each cohort (Figure 2A). A RF model built using the baseline clinical characteristics presented in Table 1 demonstrated no predictive ability and performed worse than the mean of the null distribution (Figure 2B). Models trained using the full OSU_Senescence dataset showed modest improvement, reaching at the 69^th^ percentile (Figure 2C).

To improve performance, we performed feature selection using an “all-relevant” wrapper algorithm, Boruta, and univariate logistic regression^30^. Boruta identified seven transcripts retained in the majority of 25 runs, including mRNAs associated with T cell subsets (*AHR*, *ZBTB7B*, *FOXP3*), inhibitory receptors (*HAVCR2*, *PVRIG*), cell cycle regulation (*ARF*), and co-stimulation (*CD4*). A model incorporating these features performed within the 86^th^ percentile of empirical null models (Figure 2D).

We next applied logistic regression with iterative RF modeling to rank and select the most informative transcripts. Logistic regression was performed using combined OSU_Senescence data from both cohorts to identify transcripts associated with recurrence (Figure 2E; **Supplementary Table 1**). The ten transcripts with the lowest p-values were sequentially added to RF models in order of increasing p-value, and the subset achieving the highest mean F1 score across 25 runs was selected (Figure 2F). The top-performing recurrence model included two transcripts, *CD160*, encoding a T cell regulatory receptor, and *GZMB*, encoding a cytotoxic effector molecule (mean F1 = 0.4242). This model outperformed more than 95% of empirical null models (Figure 2G). In addition, there were minimal deviations in F1 scores between runs for the model, showing model consistency between seeds (Figure 2H). Together, these efforts identify a RF model using two baseline PBTL transcripts, *CD160* and *GZMB*, as the best-performing stratifiers for predicting those who will and will not experience melanoma recurrence during adjuvant ICI monotherapy.

### A five-transcript signature provides optimal prediction of adjuvant ICI toxicity

**Figure 3:**
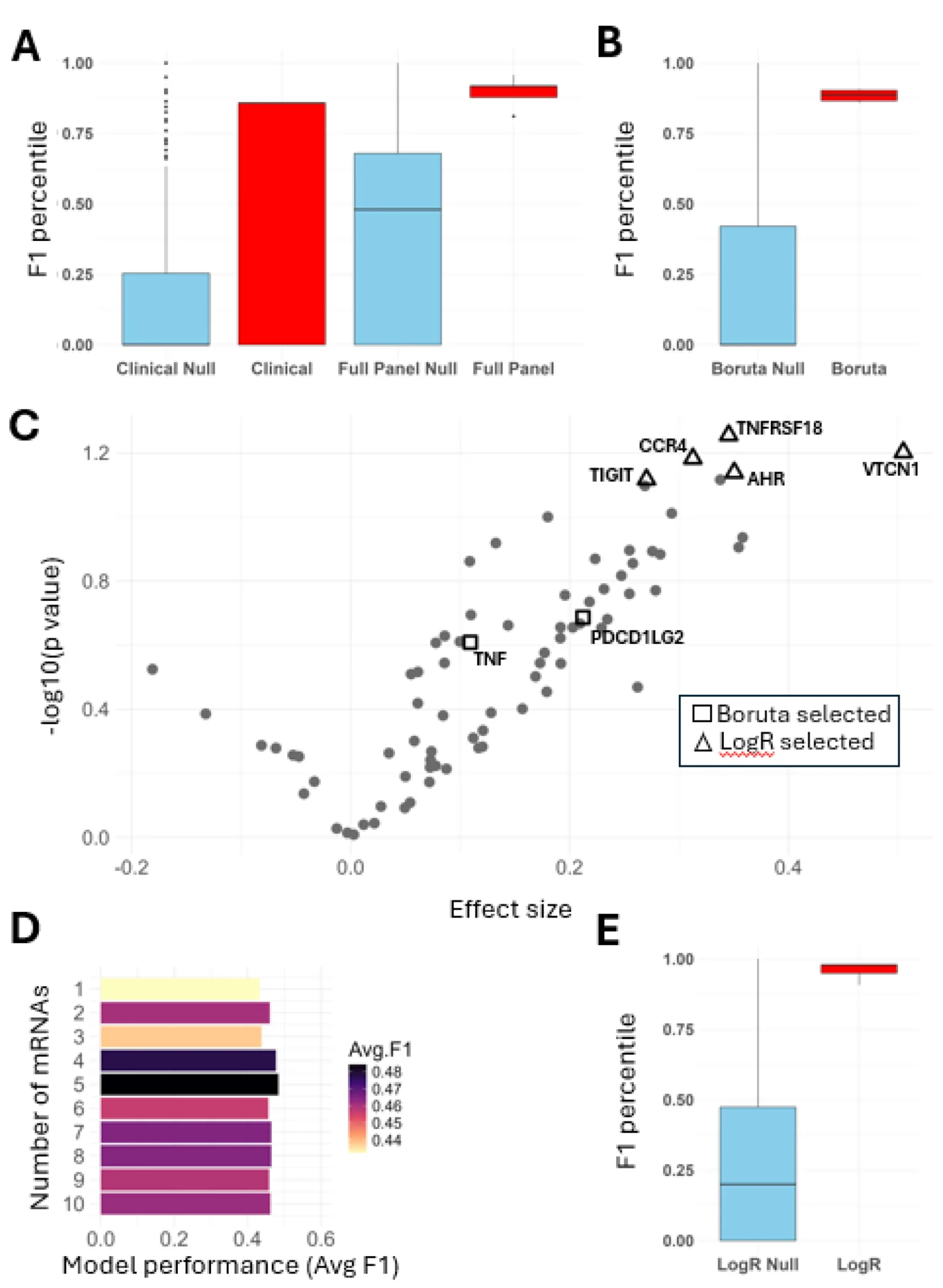
Baseline PBTL TNFRSF18, VTCN1, CCR4, AHR, and TIGITlevels identify adjuvant toxicities. (A) Percentile distribution derived from 25,000 random forest F1 scores utilizing datasets with permuted patient outcomes (blue) and 25 predictive models with unpermuted patient outcomes (red). Models utilize clinical characteristics (left), or all 79 transcripts in the OSU_Senescence panel (right). (B) Models as described in (A) based on the two transcripts identified as key features by a majority of Boruta runs. (C) Logistic regression of OSU_Senescence transcripts by outcome, with position on x axis indicating higher expression in patients experiencing toxicity (left) versus those who completed therapy (right). (D) Mean F1 scores (25 runs) for random forest models using an increasing number of mRNAs, added in order of significance (D), to predict toxicity. (E) Models as described in (A) utilizing the best performing selection of transcripts selected by logistic regression, TNFRSF18, VTCN1, CCR4, AHR, andTIGIT, as identified in (D).

We used a similar four-pronged approach to identify optimal models for predicting adjuvant ICI toxicity. The clinical characteristics model again demonstrated no predictive ability, failing to identify patients who developed toxicity (Figure 3A). In contrast, models incorporating the full OSU_Senescence panel showed improved performance, reaching the 93.4^th^ percentile relative to empirical null models (Figure 3A).

Boruta feature selection retained much of this predictive signal with only two transcripts, *TNF*, encoding a cytotoxic cytokine, and *PDCD1LG2*, encoding an inhibitory ligand. However, model performance was modestly reduced, reaching only the 89^th^ percentile (Figure 3B). Logistic regression-based feature selection identified five key transcripts associated with T cell regulation (*TNFRSF18*, *VTCN1*, *TIGIT*), trafficking (*CCR4*), and subset specification (*AHR*). A model incorporating these transcripts achieved the highest performance, reaching the 96.7^th^ percentile (Figure 3C–E; **Supplementary Table 2**). Collectively, these analyses define the highest-performing RF model for discerning patients more likely versus less likely to experience toxicity from adjuvant ICI monotherapy utilizing only five baseline transcripts (*TNFRSF18*, *VTCN1*, *TIGIT, CCR4, AHR*).

### Metastatic cohort characteristics

**Figure 4:**
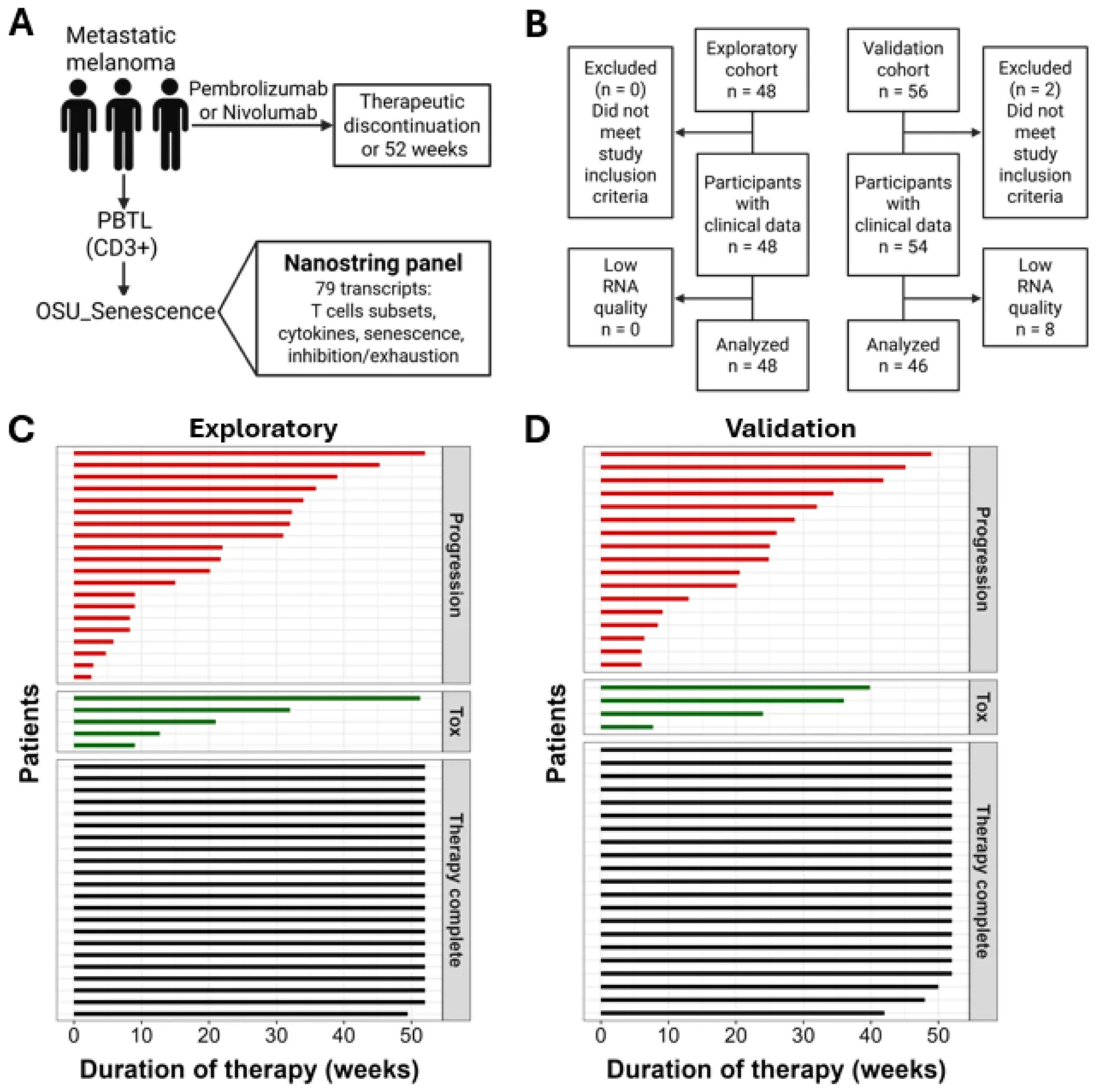
Metastatic study overview and patient timelines (A) Overview of study timeline and measured outcomes. Patients with metastatic or unresectable melanoma were enrolled prior to the initiation of ICI monotherapy. Predictive models were trained on baseline PBTL OSU_Senescence profiles, clinical, and outcome data. (B)CONSORT-style flow diagram depicting patient recruitment to the exploratory and validation cohorts with reasons for exclusion from the final analysis. (C, D) Treatment timelines for patients in the exploratory (C) and validation (D) cohorts, grouped according to clinical outcome.

In contrast to the adjuvant setting, patients with metastatic or unresectable melanoma have more advanced disease that remains clinically evident and may contribute to ongoing immune evasion. Given this biological distinction, and the limited understanding of overlap between biomarkers in the adjuvant and metastatic settings, we conducted a similar analysis of patients with metastatic or unresectable melanoma slated to receive pembrolizumab or nivolumab monotherapy (Figure 4A).

The exploratory cohort (n=48) was previously described, whereas the validation cohort included an additional 46 patients with metastatic or unresectable melanoma[35]. The cohorts were well matched with respect to sex, age, stage, and BMI (Table 2); however, LDH trended lower in the validation cohort (p=0.06), and there was a shift from pembrolizumab to nivolumab predominance, likely reflecting changes in prescribing practices (p<0.001). Recruitment, sample collection, PBTL isolation, and OSU_Senescence analysis were conducted in the same manner as in the adjuvant cohorts. Two patients were excluded due to the lack of baseline samples (Figure 4B). Patients were prospectively followed for disease progression or treatment-limiting toxicity for up to one year or until completion of therapy (Figure 4C–D).

### A single PBTL transcript, *CD45RB*, optimally predicts metastatic progression

As in the adjuvant cohorts, we developed four RF models to predict progression within one year of treatment initiation and benchmarked their performance (F1 score) against the empirical null models.

**Figure 5:**
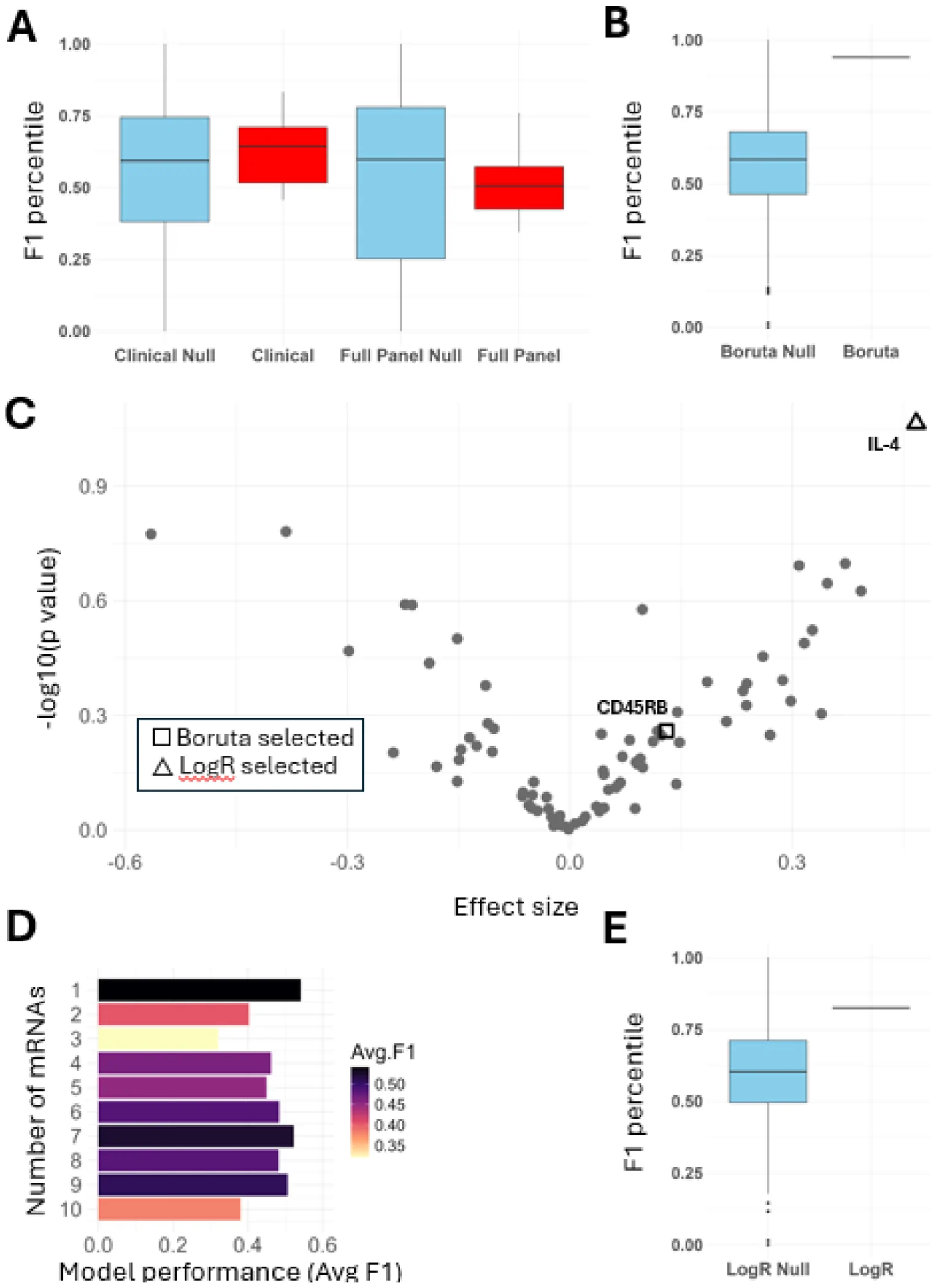
Optimal prediction of progression in metastatic patients using CD45RB (A) Percentiles of 25,000 random forest models which used datasets of permuted patient outcomes (blue) and 25 predictive models with unpermuted patient outcomes (red). Models utilize clinical characteristics (left), or all 79 transcripts in the OSU_Senescence panel (right). (B) Models as described in (A) based on the single transcript identified as a key feature by a majority of Boruta runs. (C) Logistic regression of OSU_Senescence transcripts by outcome. X axis indicates higher expression in patients who would complete one year of ICI therapy (right) versus those whose disease would progress in that time (left). (D) Mean random forest model performance (25 runs) for predicting progression, with mRNAs sequentially incorporated according to increasing p-value (C). (E)Models as described in (A) using the single mRNA identified in (C) (IL-4).

Models based on clinical characteristics alone showed limited ability to identify progression, performing at only the 64.3^rd^ percentile when comparing against empirical null models (Figure 5A). Performance was even lower for models using the full OSU_Senescence panel, which only reached the 51.2^nd^ percentile (Figure 5A).

Boruta identified a single feature, the *CD45RB* transcript, in the majority of 25 runs. RF models using this mRNA, which encodes a T cell surface protein frequently used as a marker for naïve T cells, demonstrated improved performance, reaching the 93.9^th^ percentile for identifying progression (Figure 5B). Although the best-performing logistic regression model also included a single transcript, encoding the cytokine *IL4*, its performance was lower than that of the Boruta-selected model, reaching only the 82.5^th^ percentile (Figure 5C–E; **Supplementary Table 3**). Collectively, these findings implicate a single pre-therapy PBTL mRNA, *CD45RB*, as the most important feature for discriminating patients who will experience on-therapy melanoma progression in the metastatic ICI monotherapy setting.

### Application of predictive models across the adjuvant and metastatic settings

The extent to which predictive biomarkers overlap between adjuvant and metastatic melanoma remains unclear. To address this, we constructed models trained on the exploratory cohort from one setting and tested on the validation cohort from the complementary disease setting. Each model incorporated only the top-performing features identified in our analyses of each individual cohort (e.g., *CD160* and *GZMB* for adjuvant recurrence, and *CD45RB* for metastatic progression).

**Figure 6:**
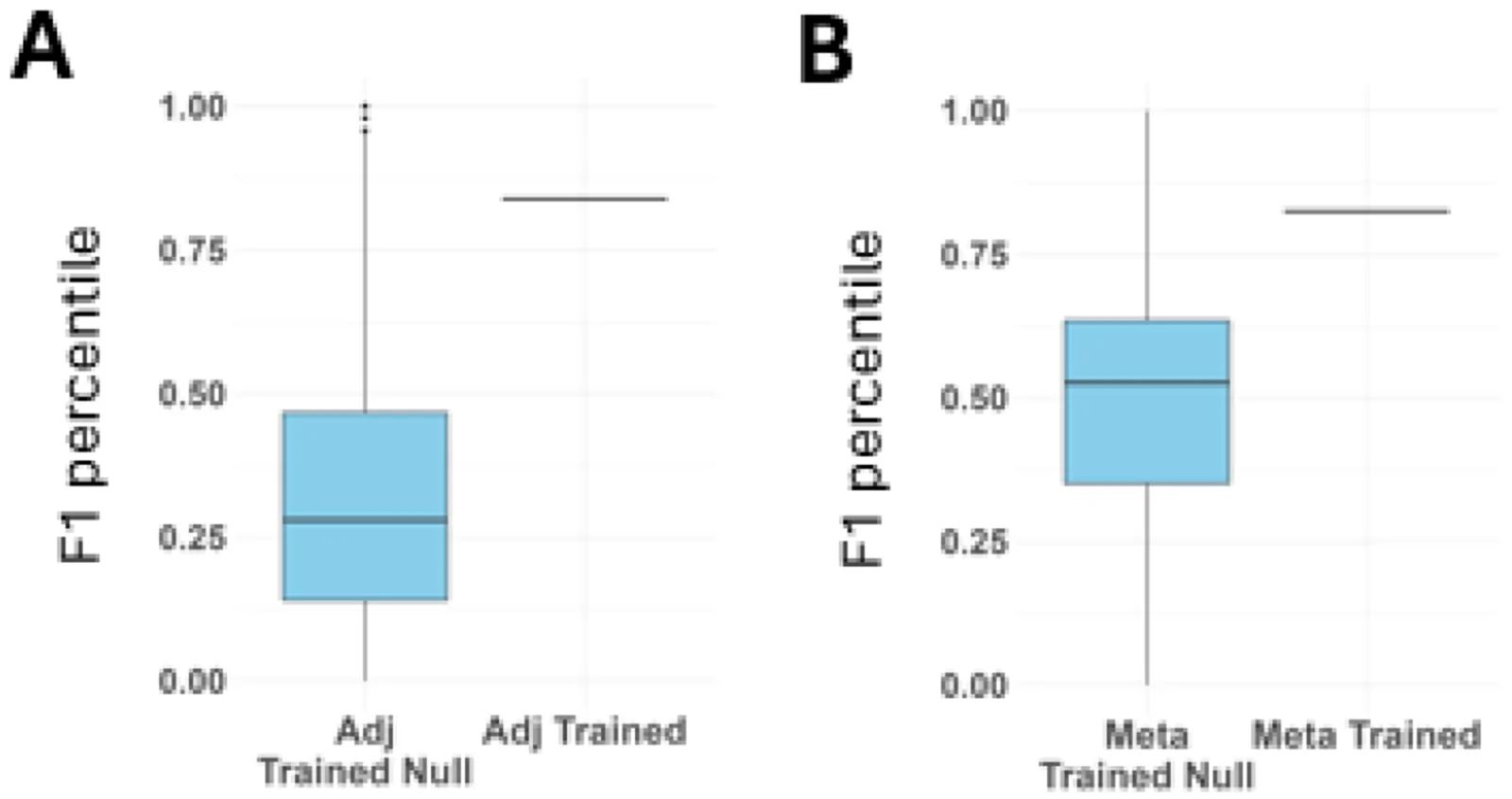
Cross adjuvant-metastatic prediction models utilizing transcripts identified by setting specific models (A-B) Percentiles of 25,000 random forest models which used datasets of permuted patient outcomes (blue) and 25 predictive models with unpermuted patient outcomes (red). Transcripts from patients that completed therapy, experienced disease recurrence, or progression were utilized. (A) Models were trained on adjuvant discovery cohort and tested on metastatic validation cohort, with both limited to transcripts of CD160and GZMB (B) trained on metastatic discovery cohort and tested on adjuvant validation cohort, with both limited to transcripts of CD45RB.

Despite being trained on distinct populations with distinct disease phenotypes, cross-trained models demonstrated moderate performance. A model trained on *CD160* and *GZMB* levels in the adjuvant cohort reached the 84.8^th^ percentile when applied to metastatic patients (Figure 6A), whereas the model trained on *CD45RB* levels in the metastatic cohort reached the 82.5^th^ percentile when applied to adjuvant patients (Figure 6B). Although neither model matched the performance of the best models within each disease setting, these results suggest some shared predictive signal across disease stages.

Toxicity prediction was also conducted between cohorts. However, due to the low number of toxicities in both metastatic cohorts, no conclusions can be drawn from these cross-setting models (**Figure S1-2; Supplementary Table 4**).

## Discussion

This study investigated whether RF models trained on T cell-specific expression profiles from PBTLs could identify patients at risk of early discontinuation of adjuvant or metastatic ICI monotherapy due to disease recurrence, progression, and treatment-limiting toxicity. We identified distinct mRNA signatures that improved prediction of adverse outcomes compared to clinical variables alone. In the adjuvant setting, *CD160* and *GZMB* were associated with recurrence, whereas *TNFRSF18*, *VTCN1*, *TIGIT*, *CCR4*, and *AHR* were associated with treatment-limiting toxicity. In the metastatic setting, *CD45RB* was associated with disease progression. Model performance was modestly reduced when applied across the adjuvant and metastatic settings, suggesting that some determinants of response are shared across diagnostic classes, whereas others may be context-specific. These findings highlight the potential of pre-treatment, blood-based NanoString profiling to offer insight into underlying T cell biology and inform clinical decision making.

The limited overlap among transcripts incorporated into models predicting recurrence, toxicity, and progression suggests that distinct biological programs underlie each outcome. In the adjuvant setting, higher pre-treatment PBTL expression of *CD160* and *GZMB* was observed in patients that completed therapy without experiencing recurrence or treatment-ending toxicity. Granzyme B is a central mediator of T cell-induced apoptosis and putative prospective biomarker of ICI efficacy in metastatic melanoma. Among patients actively receiving ICI therapy, imaging of tissue samples with GZMB probes successfully separated responders from non-responders [36]. *GZMB* expression has also demonstrated prognostic significance in adjuvant colorectal cancer, independent of the expression of other T cell effector genes [37]. CD160 is a transmembrane protein with multiple membrane-associated and soluble isoforms capable of binding Herpes Virus Entry Mediator (HVEM) to elicit context-dependent immune modulatory activity [38]. In murine models, CD160 plays a critical role in the formation of memory CD8+ T cells, and its expression is associated with resistance to terminal exhaustion, enhanced clonal expansion, stronger anti-tumor immunity, and improved response to anti-PD1 therapy [39, 40]. These findings are consistent with the idea that robust cytotoxic capacity facilitates control of residual disease following melanoma surgical resection.

In contrast, T cell transcripts associated with treatment-limiting toxicity in the adjuvant setting were enriched for immune regulatory pathways. Lower levels of checkpoint-associated transcripts (*TNFRSF18*, *VTCN1*, *TIGIT*) in patients who developed toxicity are consistent with diminished immune restraint and heightened immune activation. *AHR*, which was also reduced in T cells from patients who developed toxicity, has been implicated in regulatory T cell development and the restraint of excessive effector T-cell activity [41–43]. AHR knockdown also has been shown to improve memory and effector function while decreasing TIGIT expression in CAR-T cells [44]. Taken together, this supports the idea that circulating T cells can be used to predict patients with overactive immune states.

*CD45RB* emerged as the primary T cell transcript predictive of disease progression in the metastatic setting. A splice isoform of *CD45*, *CD45RB* is typically expressed in naïve and memory T cells and has also been linked to T cell persistence and functional capacity [45]. This was the only model where Boruta identified a selection of transcripts which outperformed the selection identified by logistic regression. *CD45RB* was not significantly different between outcomes yet was able to separate patients by outcome at a rate much higher than the empirical null model, though it was the worst performing predictive model. All of this suggests there is more to be discovered about the role of *CD45RB* as a biomarker in T cells. The relationship between *CD45RB* expression and tumor control may reflect differences in T cell differentiation state or the presence of a heathier, more functionally competent T cell population capable of more effectively controlling melanoma growth.

There is limited research directly comparing biomarkers of ICI outcomes in adjuvant and metastatic melanoma, although clinical trials have evaluated similar treatments in both settings. Notably, patients with adjuvant or metastatic melanoma experience comparable rates of treatment-related toxicity. Grade 3 or higher treatment-related adverse events occur in approximately 10-15% of patients treated with pembrolizumab and 14-16% of patients treated with nivolumab, largely independent of disease stage [4, 5, 46, 47]. In contrast, comparisons of clinical benefit across disease settings are more challenging because trials in the adjuvant and metastatic settings use different control arms and clinical endpoints. For example, in patients with resected stage III or IV melanoma, adjuvant ICI monotherapy reduces the risk of recurrence or death compared with placebo or ipilimumab, with reported hazard ratios between 0.57 and 0.65 [46, 47]. In metastatic melanoma, ICI monotherapy shows similar relative improvements in progression-free survival, with hazard ratios of approximately 0.57 – 0.69 when compared to ipilimumab [4, 5]. However, despite these comparable hazard ratios, patients treated in the adjuvant setting generally experience substantially longer intervals before recurrence than metastatic patients experience before disease progression, likely due to differences in baseline disease state [4, 5, 46, 47]. Our findings suggest that while clinical benefit may be similar across disease stages, the baseline biological factors that determine treatment outcomes are likely distinct among circulating T cells.

Several limitations of this study should be considered. First, this was a single-center study, which may limit generalizability of the predictive models to broader populations. Although we evaluated alternative machine learning models using XGBoost and SVM with the same datasets, RF showed the highest performance across outcomes [48, 49]. In addition, the study was restricted to patients receiving ICI monotherapy, which was the standard of care at the time of sample collection. Newer therapeutic agents and drug combinations could alter the biomarkers most informative for outcome prediction. The adjuvant cohort exhibited substantial class imbalance, with considerably more patients completing therapy (n=59) than experiencing recurrence (n=19) or treatment discontinuation due to toxicity (n=21). Similarly, the metastatic cohort contained relatively few toxicity events (n=9), limiting our ability to identify toxicity-associated biomarkers and to compare toxicity-associated predictors across disease stages. Furthermore, because only nucleic acids, rather than viable cells, were preserved from patient blood samples, functional studies investigating the biological origin and significance of the predictive transcripts could not be performed. The panel was also targeted in its scope but did not include every marker important for T cell differentiation and function, and clonal expansion would be difficult to investigate due to the bulk sequencing nature of the research. Furthermore, our research only investigated T cells, though it is conceivable other components of the immune system could play a role in ICI effectiveness. Finally, Common Terminology Criteria for Adverse Events (CTCAE) grades and detailed irAE classifications were not available in our dataset, limiting analyses to treatment-discontinuing toxicities rather than toxicity severity.

In conclusion, pre-treatment peripheral blood T-cell transcriptional states were associated with clinically meaningful outcomes in patients receiving ICI monotherapy for melanoma. Distinct signatures predicted recurrence, progression, and treatment-limiting toxicity, indicating that these outcomes are driven by partially nonoverlapping immune programs. The reduced transferability of models between adjuvant and metastatic disease further suggests that disease context influences the relationship between circulating immunity and therapeutic outcome. If validated prospectively, blood-based T-cell profiling could provide a minimally invasive approach for identifying patients most likely to benefit from immunotherapy and those at increased risk of toxicity before treatment initiation.

## Supplementary Material

This is a list of supplementary files associated with this preprint. Click to download.

- FigureS1S2.docx
- JMolMedSupTables.xlsx

## Figures and Tables

**Table 1:** Patient demographic and clinical characteristics by cohort for adjuvant patients

Adjuvant Cohorts	Level	Exploratory	Validation	p value
n		50	59	
Sex	F	24 (48.0%)	22 (37.3%)	0.350
	M	26 (52.0%)	37 (62.7%)	
Reason for discontinuation	Other	2 (4.0%)	8 (13.6%)	0.246
	Recurrence	10 (20.0%)	9 (15.3%)	
	Therapy complete	30 (60.0%)	29 (49.2%)	
	Toxicity	8 (16.0%)	13 (22.0%)	
Age (mean (SD))		57.22 (16.32)	59.53 (14.49)	0.436
BMI (kg/m^2^) (mean (SD))		31.41 (7.13)	31.32 (6.67)	0.944
Platelets (K/μL) (mean (SD))		231.34 (50.95)	236.95 (62.82)	0.614
LDH (U/L) (mean (SD))		151.52 (33.79)	166.05 (47.33)	0.072
Stage	I	0 (0.0%)	1 (1.7%)	0.651
	II	5 (10.0%)	4 (6.8%)	
	III	37 (74.0%)	47 (79.7%)	
	IV	8 (16.0%)	7 (11.9)	
Therapy	Nivolumab	44 (88.0%)	34 (57.6%)	0.001
	Pembrolizumab	6 (12.0%)	25 (42.4%)	

**Table 2:** Patient demographic and clinical characteristics by cohort for metastatic patients

Metastatic Cohorts	Level	Exploratory	Validation	p value
n		48	46	
Sex	F	15 (31.2%)	9 (19.6%)	0.288
	M	33 (68.8%)	37 (80.4%)	
Reason for discontinuation	Other	1 (2.1%)	4 (8.7%)	0.545
	Progression	20 (41.7%)	17 (37.0%)	
	Therapy complete	22 (45.8%)	21 (45.7%)	
	Toxicity	5 (10.4%)	4 (8.7%)	
Age (mean (SD))		67.65 (14.93)	68.87 (13.45)	0.678
BMI (kg/m2) (mean (SD))		30.17 (6.23)	30.86 (7.03)	0.616
LDH (U/L) (mean (SD))		226.00 (164.39)	174.61 (82.56)	0.060
Stage	II	1 (2.1%)	0 (0.0%)	0.297
	III	2 (4.2%)	5 (10.9%)	
	IV	45 (93.8%)	41 (89.1%)	
Therapy	Nivolumab	8 (16.7%)	35 (76.1%)	<0.001
	Pembrolizumab	40 (83.3%)	11 (23.9%)	

## Data Availability

Copies of the data sets generated and used in this study are available at https://doi.org/10.5281/zenodo.21841704. Full datasets are available on reasonable request and with IRB approval.

## Key Message

- Peripheral blood T cells provide minimally invasive predictive biomarkers
- Machine learning identified compact transcript profiles for ICI outcome prediction
- Optimal predictive signatures differ between adjuvant and metastatic melanoma
- Distinct T-cell programs may underlie outcomes across disease settings
